# Adaptive feedback control for the high‐precision management of in‐tissue drug concentrations

**DOI:** 10.1002/btm2.70146

**Published:** 2026-06-03

**Authors:** Murat Kaan Erdal, Julian Gerson, Tod E. Kippin, Kevin W. Plaxco, Joao P. Hespanha

**Affiliations:** ^1^ Department of Electrical and Computer Engineering University of California, Santa Barbara Santa Barbara California USA; ^2^ Department of Psychological & Brain Sciences University of California, Santa Barbara Santa Barbara California USA; ^3^ Department of Chemistry and Biochemistry University of California, Santa Barbara Santa Barbara California USA

**Keywords:** biosensor, electrochemical aptamer‐based (EAB) sensors, feedback‐controlled drug delivery, personalized medicine, therapeutic drug monitoring

## Abstract

The recent availability of a technology providing real‐time, seconds‐resolved in vivo drug concentration measurements has opened the door to performing fully autonomous, closed‐loop feedback control over drug dosing. The controllers employed in prior demonstrations of such dosing, however, were designed and optimized using population‐based pharmacokinetic models. In the face of individual pharmacokinetic variation (between subjects or even within a single subject over time as their physiology varies), these controllers must be set rather conservatively so as to avoid potentially dangerous overshooting. This, however, slows the speed with which they achieve the desired set point and opening the possibility of their nevertheless still overshooting if the response of a subject differs too much from that of the “average” subject. To address these issues, here we have developed an adaptive feedback‐control system that, rather than employing population‐pharmacokinetic information, instead uses real‐time drug concentration measurements to individualize drug delivery to the specific subject, “on the fly.” To achieve this, the system estimates the pharmacokinetics of the individual subject during the initial stages of the infusion, and then continuously updates this subject‐specific pharmacokinetic model to maintain effective controller performance even in the face of physiological variations brought on, for example, by changing health status. Using this approach, we then demonstrate a feedback controller that rapidly (20–30 min) achieves and accurately (5%–12% root‐mean‐squared deviations, though this also includes sensor noise) maintains pre‐defined concentrations of the anesthetic procaine in the ventricles of live rats, an application that, due to the delays associated with intracranial drug transport, represents a particular challenge for feedback‐controlled intravenous drug delivery. Given the precision and accuracy it achieves in our rat animal model, we believe that the use of adaptive feedback control will ultimately enable safer, more precise drug dosing in humans.

## Graphical abstract

We describe an adaptive feedback‐control system that autonomously models the site‐specific pharmacokinetics of a target molecule in each individual subject and adjusts the rate with which the molecule is delivered according to model predictions. This approach can enable precise and safer drug delivery.

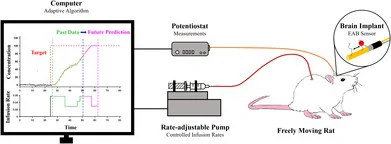

**Translational Impact Statement**

Achieving personalized dosing, and the safer, more effective drug delivery this provides, requires anticipating how each patient will respond to a drug, including drug disposition at the site of action. Advancing on this idea, here we demonstrate an adaptive feedback controller that uses real‐time drug measurements collected at the site of action to learn the individual's pharmacokinetics on‐the‐fly, and then uses this information to actively maintain stable drug levels. Applied to controlling anesthetic concentrations in the brains of live rats, the approach enables fully‐autonomous, high‐precision personalized drug dosing. By ensuring accurate and responsive drug administration, this technology could enhance safety and efficacy of drug delivery in a range of clinical settings.

## 1 INTRODUCTION

The goal of therapeutic drug monitoring (TDM) is to control in vivo drug concentrations so as to achieve optimal clinical outcomes. This has traditionally been performed by manually adjusting dosing based on laboratory measurements of blood samples drawn intermittently over the course of repeated treatments and informed by knowledge of the average pharmacokinetics of the drug in the relevant patient population and, sometimes, the patient's specific clinical characteristics.^1^, ^2^, ^3^, ^4^ Unfortunately, however, the slow, cumbersome nature of laboratory‐based drug measurements and the sometimes poor fit of population average pharmacokinetics to any specific, individual patient has historically rendered TDM imprecise, costly, and labor‐intensive, limiting its use^5^ and eliminating its ability to provide any guidance at all regarding the first drug dose. In sharp contrast, a high degree of “personalization” in dosage adjustment is achieved for the treatment of Type‐1 diabetes via the combination of real‐time, in vivo glucose measurements and an insulin pump. Working together with a control algorithm, this system has been shown to improve the time blood glucose levels remain within the normal range, improving the quality of care for patients.^6^, ^7^ But because the enzymatic glucose sensor employed in this system has proven difficult to adapt to other analytes, it has not historically proven possible to generalize this system to the delivery of therapeutic drugs beyond insulin. However, the recent development of a platform technology, termed electrochemical aptamer‐based (EAB) sensors,^8^ supporting the continuous (i.e., at frequencies much faster than physiological timescales), real‐time measurement of in vivo drug concentrations provides a route to extending similarly closed‐loop feedback control over therapeutics beyond insulin.^9^

Comprised of a redox‐reporter‐modified aptamer (a DNA or RNA molecule selected in vitro to bind a specific molecular target) covalently attached to an interrogating electrode (Figure 1a), EAB sensors employ a binding‐linked conformational change to generate an electrochemical output easily measurable using a range of electrochemical approaches (Figure 1b). This conformation‐linked signal transduction mechanism renders EAB sensors reagentless, single‐step, and rapidly reversible, and selective enough to deploy in situ in the living body. It also renders them independent of the chemical or enzymatic reactivity of their target, thus making the platform easily adaptable to new targets. Building on this, EAB sensors have been reported against dozens of molecular targets,^14^ more than a dozen of which have been measured in situ in live rats. The latter includes second‐ or even sub‐second resolved, real‐time measurements performed in the vein (i.e., in blood plasma),^12^, ^15^, ^16^, ^17^ in the brain (the cerebrospinal fluid of the hippocampus and lateral ventricles),^10^, ^11^, ^18^ and in the solid peripheral tissues (the interstitial fluid of tumors, muscle, and the subcutaneous space).^19^, ^20^, ^21^ By providing continuous, real‐time information regarding in vivo drug concentrations, EAB sensors are uniquely well‐suited for performing closed‐loop feedback control over drug and metabolite levels in the body (Figure 1c).

**FIGURE 1:**
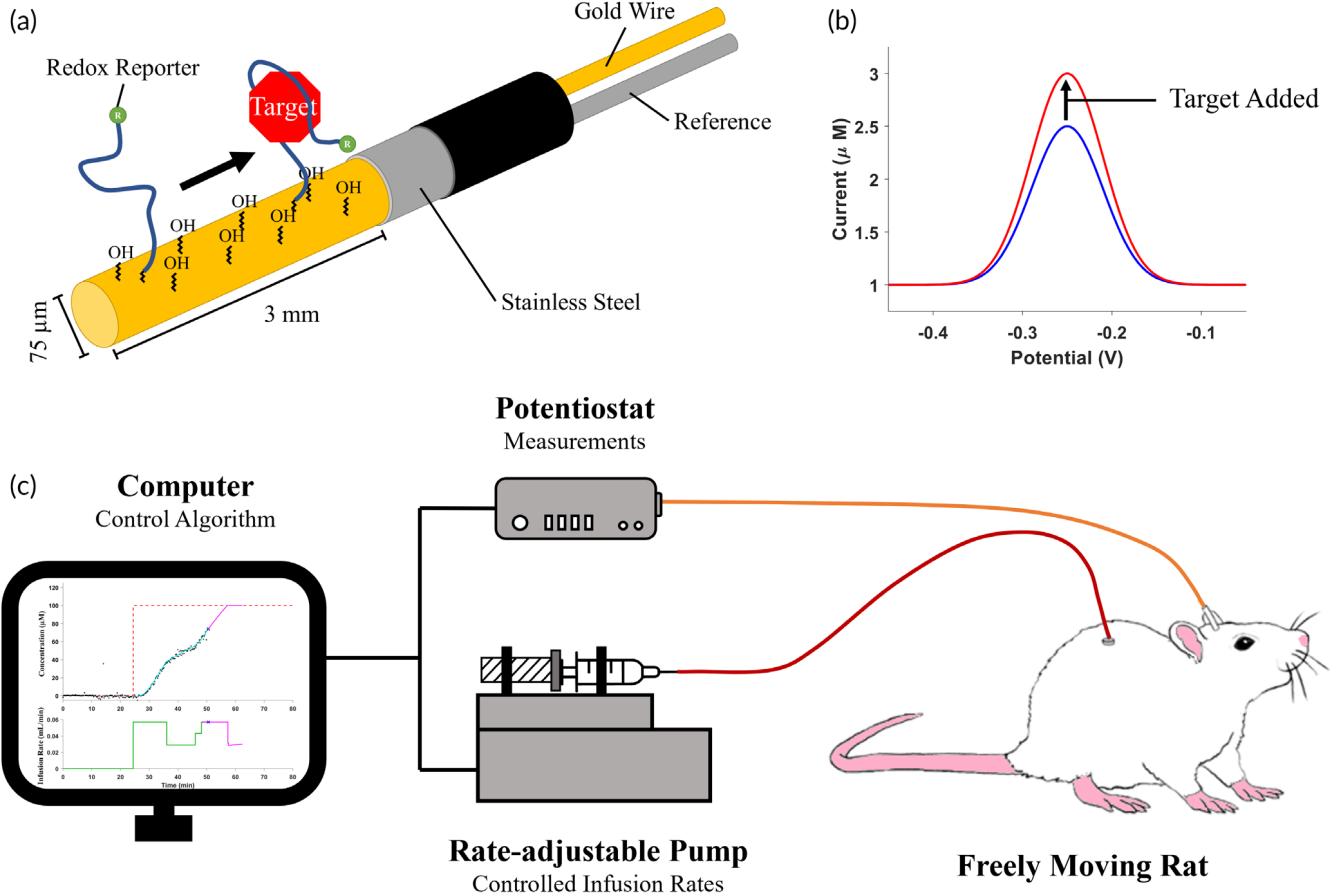
Electrochemical aptamer‐based (EAB) sensors and the experimental setup. EAB sensors support the real‐time, seconds‐resolved measurement of specific molecules in situ in the living body. (a) When designed for placement in the brain, such sensors consist of a gold working electrode and a stainless‐steel canula that is used as a counter and pseudoreference electrode.^10^, ^11^ The working electrode is functionalized by covalently attaching a redox‐reporter‐modified aptamer (a DNA sequence selected in vitro to bind to a specific target) that undergoes a conformational change upon target binding. (b) This, in turn, causes a change in peak current when the sensor is interrogated using any of a number of electrochemical approaches, including, as used here, square wave voltammetry. (c) The real‐time concentration measurements provided by EAB sensors support closed‐loop feedback‐controlled drug delivery.^11^, ^12^, ^13^ Here, we demonstrate the use of adaptive feedback control to more rapidly achieve the desired concentration of a specific molecule, using as our test bed feedback control over in‐brain drug levels, which is complicated by slow transport dynamics that themselves exhibit significant subject‐to‐subject variability.

To demonstrate the translational potential of EAB‐sensor‐informed, feedback‐controlled drug delivery, we have previously^10^, ^12^, ^13^ demonstrated the approach using rats as our model species. We used rats due to their human‐like circulatory and elimination systems and their manageable surgical scale. That is, because, as mammals, rats' pharmacokinetics, circulatory system, metabolism, bodily fluid composition, body temperature, and excretory system are relatively similar to those of humans, and, unlike mice, rats are of sufficient size for the required surgical procedures. Rats are also a widely utilized animal model in pharmacokinetic studies, due to their metabolic similarity to humans. This said, because rat body volume is orders of magnitude smaller than that of humans, and because rats typically metabolize and excrete drugs much more rapidly than humans, using feedback control to maintain constant drug levels in a rat, in general, should be more difficult than performing the same control in humans. That is, the faster time scales, due to the faster excretion and smaller distribution volumes, amplify the effect of any delays in sensing and actuation and, consequently, increase overshoot and potentially compromise stability. We note, too, that, unlike animal studies of drug efficacy, in which the drug target differs between rats and humans (due to evolutionary drift), in feedback‐control studies, we are measuring and controlling the same molecule in the rat as would be measured and controlled in humans, further decreasing the likelihood of failure in translating from rats to humans.

In our first proof‐of‐concept studies, we used a simple proportional‐integral‐derivative (PID) feedback controller informed by in vivo EAB sensor measurements to achieve the high‐precision control of plasma levels of the antibiotics tobramycin^12^ and vancomycin.^13^ Specifically, using this approach to control intravenous infusions, we demonstrated the ability to maintain both constant plasma drug concentrations as well as plasma drug concentrations that accurately follow pre‐defined, time‐varying profiles, profiles that mimic human pharmacokinetics in a rat.^12^ This ability to accurately control plasma drug and metabolite concentrations has the potential to profoundly impact both biomedical research and clinical practice.^22^ The ability to precisely control in vivo drug concentrations in animal models, for example, could improve the accuracy and efficiency of the preclinical stages of drug development by eliminating pharmacokinetic differences between animals and by enabling the accurate emulation of human pharmacokinetics in animal models.^12^ And by ensuring that drug concentrations remain within the therapeutic window, thus avoiding both undertreatment and toxicity, such a technology could likewise improve the efficiency and the safety of pharmaceutical treatments.

While our ability to control plasma drug concentrations is now established, it is the solid tissues, and not the plasma, that is the site of action of most drugs. Fortunately, the ability of EAB sensors to perform measurements throughout the body also opens the door to controlling drug concentrations in solid tissues. In the clinic, such a technology could similarly be used to maintain control over the concentration of, for example, a chemotherapeutic in a tumor or an antibiotic at a site of infection. As the first example of such control, we recently used a PID controller to achieve feedback control over intracranial concentrations of the anesthetic procaine using a sensor placed in the lateral ventricle of a live rat and intravenous delivery of the drug.^10^ The relatively slow intercompartmental transport of drugs, however, complicates feedback control in compartments other than the compartment to which the drug is being delivered. That is, when the site of delivery of the drug, such as into the vein (plasma), and the site of measurement, such as in the brain, differ, the finite rate of transport between the two renders the achievement of accurate feedback control more complex. This obstacle, the magnitude of which will vary depending on the physiological status of the subject (such as kidney, liver, or cardiac function, diet, or the presence of cross‐reacting drugs), the precise tissue site under control, and the physicochemical and pharmacological properties of the drug, renders it challenging to design effective PID controllers. Indeed, even when the site of drug measurement and delivery are both in the plasma, it is exceptionally difficult to tune PID controllers so as to avoid a large overshoot of their desired set point without being excessively slow at reaching that set point.^12^, ^13^ Worse still, subject‐to‐subject pharmacokinetic variability can cause otherwise well‐tuned controllers to perform sub‐optimally if a given subject's pharmacokinetics vary significantly from the population‐average pharmacokinetics employed to tune the controller.

In response to the limitations of PID‐based feedback‐controlled drug delivery, we have developed an adaptive feedback control algorithm that autonomously models the pharmacokinetics of the target molecule at the site of measurement in the individual subject and then, using the predictions of this highly individualized model, adjusts the rate with which the molecule is delivered. While we recently used a version of this feedback controller to maintain constant plasma drug concentrations following intravenous drug delivery,^23^ here we present the first detailed description of the algorithm and present new results for its utility in improving the speed and accuracy of control over in‐brain drug concentrations, which, due to significant transportation lags between the site of delivery (the plasma) and the site of measurement (here the cerebrospinal fluid) is a much more challenging use case. Specifically, as the test bed for this study, we employed control over intracranial procaine in live, freely moving rats.

## 2 RESULTS AND DISCUSSION

### 2.1 Limitations of population‐based PID controller

A trade‐off between performance and safety inherent in PID controllers renders their performance in feedback‐controlled drug delivery suboptimal. Specifically, controllers tuned to rapidly rise to the set point have a tendency to overshoot, particularly when the system exhibits significant lag times between the input (here drug delivery) and the resulting change in output (here drug concentration). To achieve a good balance between these effects, the parameters defining the controller are generally tuned based on the expected behavior of the system they are controlling.^24^ In the case of drug delivery, however, accurate knowledge of the pharmacokinetics of any given subject is unlikely to be available prior to the start of treatment, a problem magnified by any number of disease states that may alter drug metabolism or excretion. This can become a particularly serious problem if the tolerance for overshooting the targeted concentration is low (e.g., due to toxicity) and/or, as is the case for the control of drug concentrations in the solid tissues, the drug's transport dynamics introduce unknown delays between dosing (e.g., delivery to the blood) and the site under control. In our prior demonstration of control over concentrations of the anesthetic procaine in the rat brain,^10^ for example, significant overshooting could have lethal consequences, and the system exhibits significant delays due to the slow transport of drugs across the blood–brain‐barrier.

To account for the above‐described issues, in our prior work we used a “population approach” to generate expected pharmacokinetics driving a relatively conservative PID controller. To achieve this, we collected EAB‐sensor‐derived procaine concentration time courses following intravenous procaine dosing with a 20 min constant‐infusion while monitoring the resulting rise and fall of the drug's concentration in the brain from seven subjects (Figures 2a and S1). Then using simulations, we designed a PID controller that would have performed well (if somewhat slowly) for all seven of these animals despite the range of pharmacokinetics they exhibited. When we applied the resulting controller to an eighth animal, it successfully maintained the concentration of in‐brain procaine at the targeted 100 μM for 1.66 h with a root mean squared deviation (RMSD) of 18 μM. This performance (i.e., maintaining the in‐brain concentration within 20% of the targeted concentration) already reflects far higher precision control over in vivo drug concentrations than can be achieved using current dosing regimens^25^ which, of course, are not guided by meaningfully real‐time measurements. Nevertheless, our population‐based PID controller is not without potentially significant limitations. The population‐based controller we employed will act anomalously or even fail outright, for example, if the pharmacokinetics of the subject under control fall significantly outside of the range of pharmacokinetics observed in the test population. To avoid this, we tuned the PID controller conservatively. The downside of doing so, however, is that it required almost 1 h to reach the set‐point concentration (Figure 2b). Moreover, despite this conservativism, we can still see the danger associated with this population approach in our prior experiments. Specifically, the drug concentration in this experiment initially lagged behind the concentrations the population pharmacokinetics would predict, before then rising to the targeted concentration unexpectedly rapidly (Figure 2c). This might be due to a simple case of our subject being an outlier whose pharmacokinetics are not adequately captured by the seven animals in our training set, or because the constant, 20 min infusions we employed in our training runs do not provide sufficiently detailed information regarding the drug's pharmacokinetics in rats. More generally, even were we to run a subject that was in the training set, we need to be conservative with the PID controller design to avoid overshooting. For example, if we design the controller so that the population‐average reaches the 95% of the targeted concentration in 20 min, some of the subjects in our training‐set would have seen overshooting (Figure 2d). The problems associated with predicting the pharmacokinetics of any one individual using population data thus clearly limit the performance of PID controllers in drug delivery.

**FIGURE 2:**
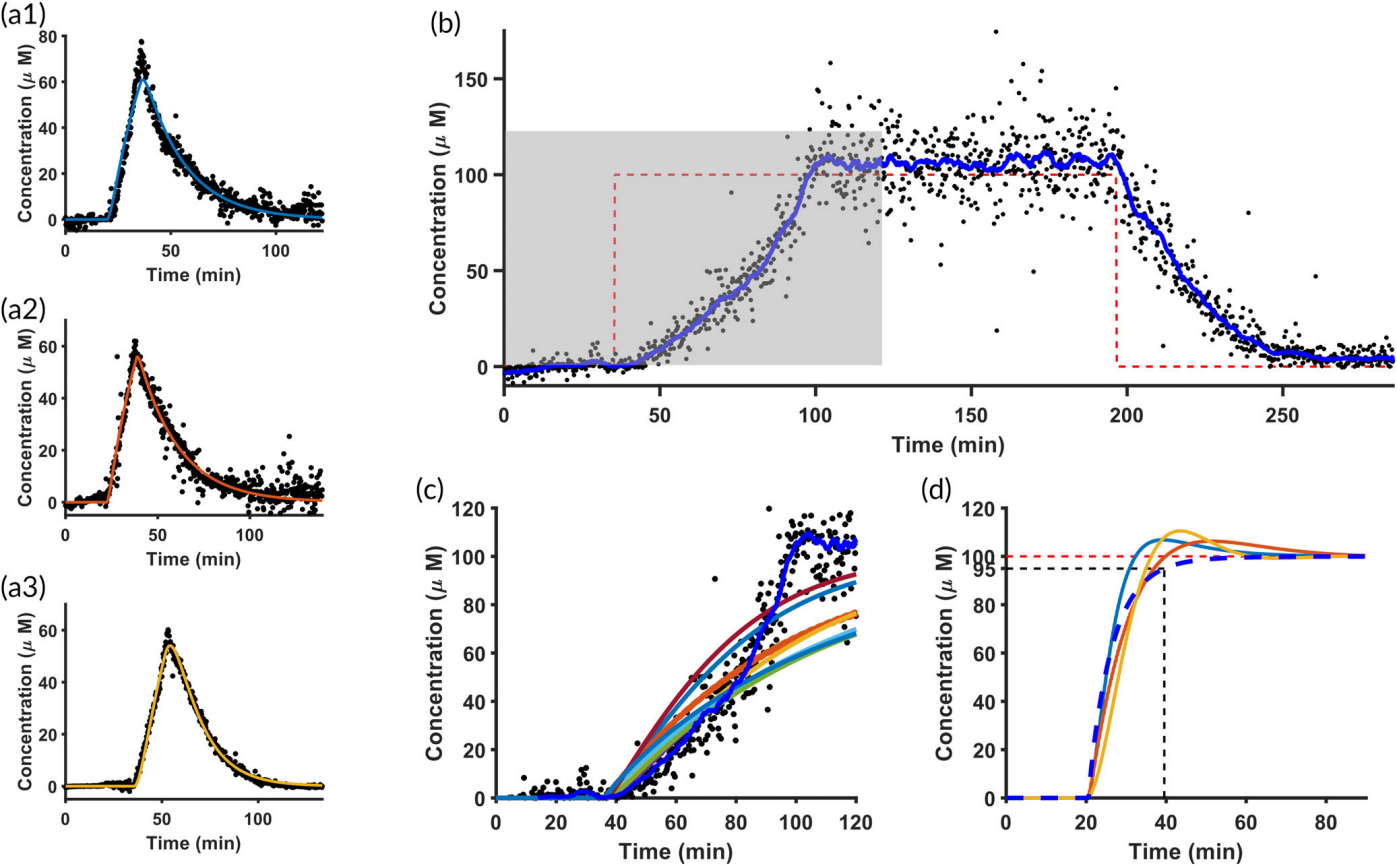
Analysis of past proportional‐integral‐derivative (PID) controlled in‐brain drug concentrations. In our prior efforts to perform feedback control over in‐brain drug concentrations, we used a PID controller that was designed using information derived from population pharmacokinetics. (a) That is, we used uncontrolled, 20‐min drug infusions to experimentally measure seven pharmacokinetic data sets (three are shown here; for the rest, see Figure SI‐1). We then designed a PID controller that, as determined via simulations employing the pharmacokinetics observed in these seven data sets, would have achieved the targeted concentration in all of them without significant overshooting in any. (b) Employing this in an eighth, out‐of‐training‐set animal, the controller reached and accurately maintained (to an RMSD of ±18 μM) the desired, 100 μM set point (the red dashed line). But because this PID controller does not accurately reflect the pharmacokinetics of any individual animal, it must be tuned rather conservatively. As a result, it required nearly 1 h to reach the desired set point. (c) Subject‐to‐subject pharmacokinetic variability can also lead to unexpected behavior when using a population‐based controller. To illustrate this, here we present the simulated responses of rats from previous experiments during the rising phase of the actual experiment (highlighted in panel b), when the difference is clearest. The trend line in the feedback control experiment (blue) at first reacts more slowly and then ramps up more quickly than all the previously observed behavior, demonstrating that the pharmacokinetics of this animal were poorly captured by our population‐based model. (d) While the population‐based controller can be tuned to deliver rapid attainment of the target concentration for the population average (blue dashed line), it often overshoots in individuals whose pharmacokinetics stray from the mean, even if, as shown here, those same individual's pharmacokinetics are part of the training model. The data sets in panel (a) are plotted with the same colors as the simulated responses in panels (c) and (d) and in Figure SI‐1.

### 2.2 Adaptive feedback controller

As a solution to the problems inherent in population‐based feedback control over in vivo drug concentrations, here we have developed an “adaptive control” approach that entirely eschews the use of population data or, indeed, any prior knowledge of the subject's pharmacokinetics. Instead, the feedback control algorithm actively determines the pharmacokinetics of each individual subject “on‐the‐fly,” while the controller is adjusting the drug delivery rate to achieve the desired drug concentration profile. As our test bed for these studies, we used the same procaine‐detecting EAB sensor we employed in our prior in‐brain feedback control study with population‐based PID controller.^10^ We inserted this into the right lateral ventricles of live rats through a 19G cannula that was stereotaxically implanted and cemented to the skull at least 1 week prior to the experiment. The sensor was then connected to the potentiostat via a cage‐top swivel. For dosing the drug, we employed a catheter that runs through the same swivel to a syringe pump, the pumping rate of which is modulated by the feedback controller.

Our adaptive controller must predict how the drug concentration at the site of control will respond to changes in the rate with which the drug is injected into the body. To achieve this without assuming any foreknowledge of the subject's pharmacokinetics (e.g., the population‐based constraints employed in our prior study), we assume that the future drug concentrations can be predicted sufficiently well by a combination of recent past values of itself and the infusion rates (Equation 1). This type of model, which are called autoregressive moving average (ARMA) models, is a common way to explore relations hidden in time‐series data sets.^26^ Under a general ARMA model, the concentration measured at the site of control at timestep $t,\;C\left(t\right),$ is assumed to be given by the following relation.

(1) $$C\left(t\right)=\sum_{i=1}^{m}\alpha_{i}C\left(t-i\right)+\sum_{i=0}^{n}\beta_{i}u\left(t-i-1\right),$$

where $\alpha_{i}$ and $\beta_{i}$ are the model parameters, and $u\left(t\right)$ is the drug infusion rate at the timestep t. Such an ARMA model is said to be of order $\left(m,n\right)$. Here we employ an ARMA model of order (2, 1), which is simple enough to ensure rapid fitting and optimization but also complex enough to capture previously observed, single‐exponential pharmacokinetics of in‐brain procaine.^10^

The ARMA (2, 1) model is sufficient to predict future in‐brain procaine concentrations with reasonable accuracy even when the concentrations are being actively driven by a controller. To show this, we used the data from our previous, population‐based PID experiment (Figure 2b). At every time point in this data set (starting after about 1 h to allow for capturing a nonzero response), we fitted the ARMA (2, 1) model to the 100 most recent concentration measurements and the corresponding drug infusion information, and simulated the next 50 timesteps using the fitted model and the future dosing that, originally, had been determined by the PID controller. Doing so, we find that, based on knowledge of the current concentration, prior concentrations, and the infusion profile that produced these concentrations, our ARMA (2, 1) model was able to predict future drug concentrations (i.e., the concentration looking 50 timesteps forward) with acceptably good accuracy (Figure 3a). Specifically, these “50‐timesteps forward” predictions captured approximately 78% of the noisy measurements within the estimated 95% confidence intervals (Figure 3b). We believe this is an acceptably good accuracy to show the predictive capability of ARMA (2, 1) model considering the simplifying assumptions on the noise characteristics and pharmacokinetic modeling. This ability to predict future concentrations forms the basis of our new, adaptive controller.

**FIGURE 3:**
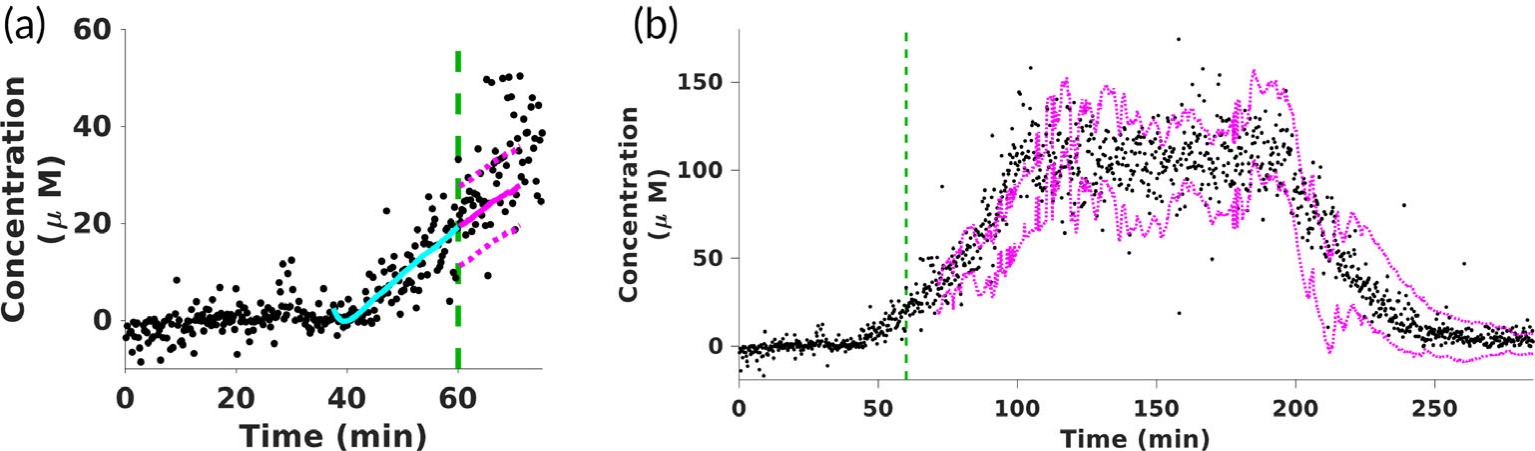
Performance of autoregressive moving average (ARMA) (2, 1) model. Here, we demonstrate that an ARMA (2, 1) model (as opposed to a higher order or more complex ARMA model) is sufficient for our needs by showing the compatibility of such a model with the results of a previous, proportional‐integral‐derivative (PID)‐controller‐based, in‐brain control experiment. (a) Specifically, at about 60 min (dashed green line), we have fit the ARMA (2, 1) model (light‐blue line) to the prior 100 measurements and used this fitted model to predict the concentrations expected over the next 50 measurements (magenta line). The large majority of then‐measured concentrations (black dots) fall within the estimated 95% confidence intervals (defined by the magenta dotted lines) of the ARMA (2, 1) model prediction. (b) Propagating this approach forward, we find that the model continues to accurately predict the concentrations observed 50 measurements ahead throughout the entire PID control experiment. Specifically, 78% of the observations fall within the estimated 95% confidence intervals around ARMA (2, 1) model prediction (dotted lines), with the discrepancy between 78% and 95% presumably arising due to sensor noise and variations in the animal's physiology (i.e., changes in the true pharmacokinetics).

The adaptive feedback algorithm estimates the individual subject's pharmacokinetics (determines the coefficients of our ARMA model) by beginning the feedback control with an initial, “learning phase” infusion profile and then observing how the drug concentration at the site of control varies in response to this initial infusion. We designed the learning‐phase infusion profile, which is defined by the same set of rules for all subjects (Figure 4), so as to enhance the speed with which the algorithm converges on an accurate, subject‐specific pharmacokinetic model while also rapidly moving toward the targeted concentration. Specifically, we designed it as a “impulse‐like” infusion profile similar to those that we have previously shown are optimal for estimating pharmacokinetics.^27^ This starts by delivering the drug at the (clinically‐, technically‐, or safety‐and‐welfare‐defined) maximum infusion rate until the measured concentration reaches a pre‐defined, intermediate value; here we set this to 30%–40% of the desired final set point ($C_{1}$ in Figure 4 and Algorithm S1‐1). The infusion rate is then reduced by half until the plasma concentration reaches a second predefined, intermediate value; here set to 60%–70% of the final set point ($C_{2}$ in Figure 4 and Algorithm S1‐1). If this threshold is not achieved after a certain amount of time the infusion rate is gradually increased until the threshold can be achieved (Figure 4 and Algorithm S1‐1). By monitoring the subject's response to this stepped initial infusion profile, the control algorithm derives, in real time, an “individualized” pharmacokinetic model for the subject (and tissue site) under control.

**FIGURE 4:**
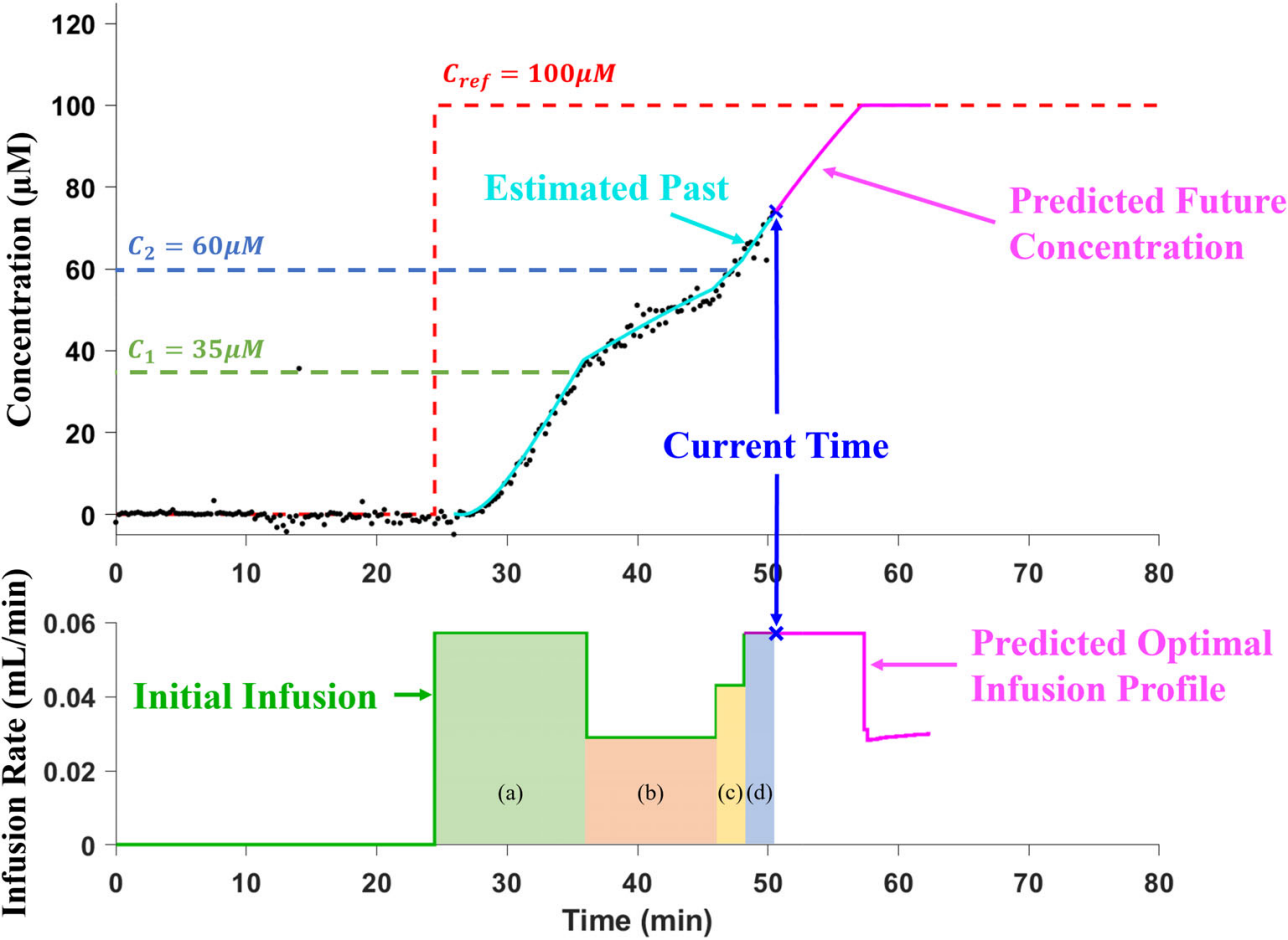
A snapshot of the adaptive control algorithm at work. The adaptive feedback control algorithm we employ has four phases. In the first, the controller delivers the drug at the maximum‐allowed (by the equipment, by physiological concerns, or by other animal welfare concerns) infusion rate (the green region [a]). Once the concentration reaches the threshold $C_{1}$, the controller then (phase 2) reduces the delivery rate to a rate that is a predetermined percentage, 50% here, of the maximum rate (the orange region [b]). When a second threshold concentration value, $C_{2}$, is reached, control is then passed (phase 3) to the adaptive controller (the blue region [d]). Sometimes, however, reaching $C_{2}$ with the reduced infusion rate is not possible, in which case the controller increases the infusion (phase 2A) rate gradually until the threshold $C_{2}$ can be reached (the yellow region [c]). Once the concentration reaches to $C_{2}$, the adaptive controller defines all further infusions. As its first step, the system identification algorithm identifies the pharmacokinetic model that best describes the recently observed concentration data (the light blue line in the top panel) and the corresponding infusion rate history (green line for the initialization‐phase and purple line for the adaptive‐feedback history). In the next step, model predictive control algorithm finds the optimal infusion profile (the magenta lines in the lower panel) to drive the concentration to its targeted value as rapidly as possible given the identified model in the first step (magenta line, upper panel). At each new measurement, the pharmacokinetic model is updated and the cycle is repeated.

Once the measured drug concentration reaches the second intermediate concentration, the controller shifts from the pre‐defined, learning‐phase infusion profile to an actively controlled infusion profile optimized to achieve and maintain the desired set point concentration (Figure 4 and Algorithm S1‐1). Specifically, the controller uses the ARMA (2, 1) model that best fits the concentration profile measured during the initial infusion stage to predict how future concentrations will vary for any given infusion rate. However, since this model is an approximation of the animal's true pharmacokinetics, we cannot trust it to provide reliable predictions arbitrarily far into the future. For example, the rate “constants” describing drug transport or elimination, which are under physiological control, can vary with time.^28^ To circumvent such effects, the algorithm continues to update its subject‐specific pharmacokinetic model after each additional concentration measurement throughout the period of control.

To achieve the targeted concentration, the controller employs a model predictive control mechanism^29^ based on the model identified in the first stage. For a detailed review of similar approaches, we refer to a recent paper by Hewing and coworkers.^30^ The idea behind model predictive control is to find the optimal infusion profile that will achieve the desired set point concentration for the given model description. However, since the model identified in the first stage is not necessarily an accurate model of the subject's true pharmacokinetics (no matter how well it describes the recently observed concentration measurements), the controller only uses the first value of the optimal infusion profile at the time. At the next time interval, the next newly measured concentration (and the corresponding last used infusion rate) is added to the data set while the oldest data pair (the paired concentration measurement and infusion rate) is removed from consideration, and the process is repeated again (Algorithm S1‐1).

A key set of parameters in our adaptive control algorithm is the threshold values defining when to switch control over drug delivery from the pre‐defined learning phase infusion profile to active feedback control ($C_{1},C_{2}$ in Figure 4 and Algorithm S1‐1). These values should be chosen based on (i) how rapidly the targeted concentration need be achieved and (ii) the expected noise in the measurements. For example, in our first attempt, for which we set the targeted concentration at 100 μM, we set the threshold to lower the infusion rate from the maximum rate to half‐maximum, $C_{1}$, to 35μM and the threshold to start active control, $C_{2}$, to 60μM (Figure 4). This said, here we employed relatively conservative thresholds (i.e., thresholds significantly below the targeted concentration) in order to prevent build‐up of the drug to a level at which the adaptive controller cannot avoid overshooting the set point. If, in contrast, we expected the drug concentration to rise much more slowly (or rapidly), we could have set these higher (or lower).

The adaptive controller rapidly reaches and accurately maintains in‐brain procaine concentrations at a pre‐defined, set concentration (Figure 5). For example, using this approach we challenged the adaptive controller to hold in‐brain procaine concentrations at 100 μM for 1 h. After an approximately 24 min learning phase following the start of infusion, the controller took over and achieved its target concentration some 7.5 min later (i.e., 31.5 min after the start of infusion). It then held this concentration with an RMSD of 10.2μM (Figure 5a). Both of these values (i.e., the time‐to‐set point and the control precision) represent approximately two‐fold improvements over the performance of our earlier, PID‐based controller.^10^ And, again: the algorithm achieved this improvement without employing any prior knowledge of the drug's pharmacokinetics at either the population level (i.e., unlike the PID controller, which uses such information) or at the level of the individual subject. Repeating the fixed‐concentration control experiment with a set point of 200μM, the adaptive controller assumed control at 12.4 min after the start of drug infusion and achieved its target concentration some 9.7 min later (i.e., 22.1 min after the start of the infusion). It then maintained the targeted concentration (RMSD of 11.3 μM) until control was released after about 1 h (Figure 5b), values that, once again, reflect a significant improvement over the PID controller.

**FIGURE 5:**
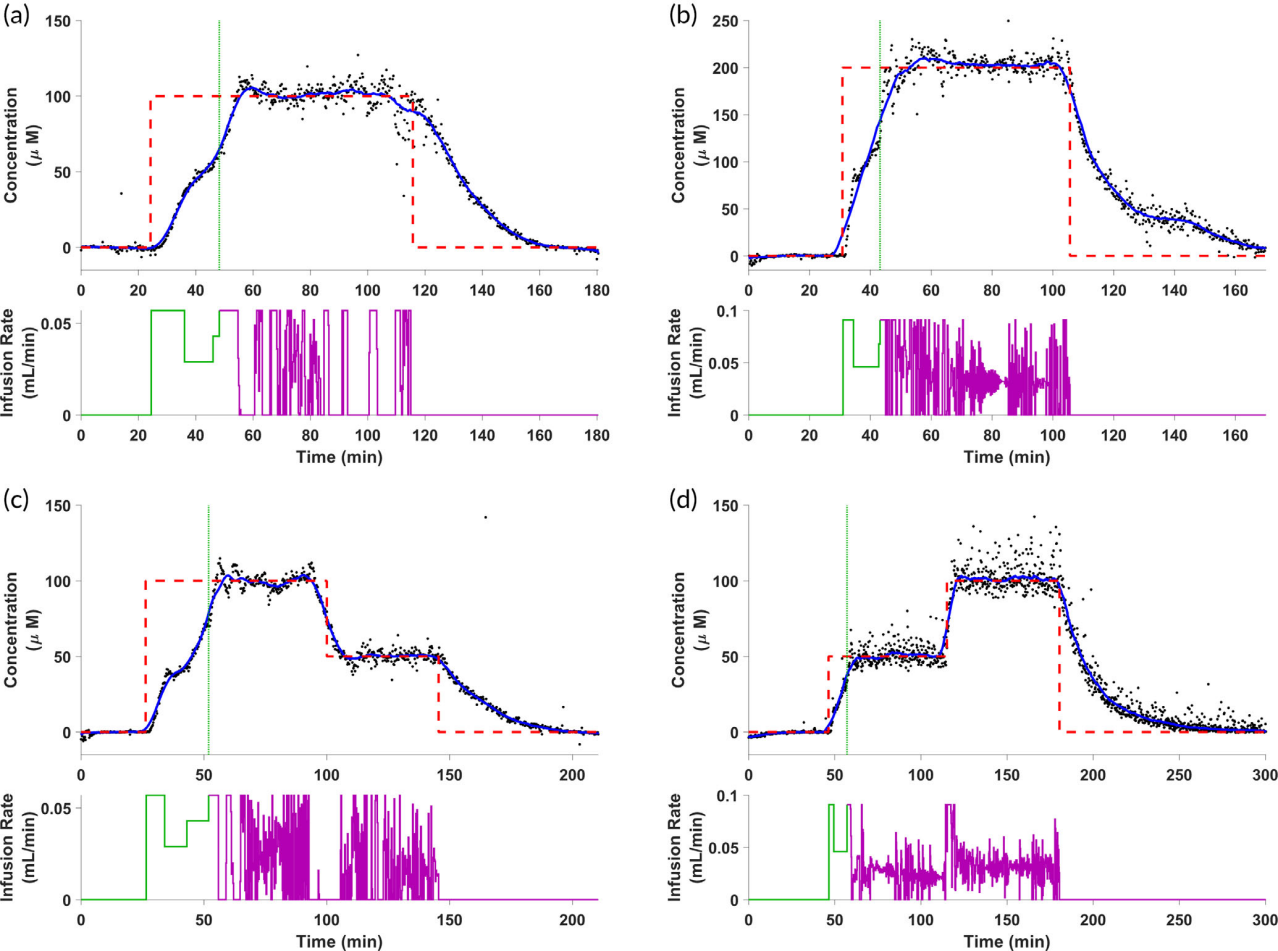
Application to different target concentration profiles. Adaptive control rapidly achieves and precisely maintains its targeted concentration profile. (a) Here, for example, we used our algorithm to achieve and remain at a procaine level of 100 μM for about 1 h with an root mean squared deviation (RMSD) of 10.2 μM. (b) Next, we repeated the prior experiment in a second animal with a targeted concentration of 200 μM. We then held this concentration (with an RMSD value of 11.3 μM) for about 1 h. (c, d) After establishing that the algorithm can maintain constant concentrations, we next used it to achieve pre‐defined, time varying concentration profiles in which we stepped the targeted concentration from high to low (c) and from low to high (d). For each data set we highlighted the point (green lines) at which the initialization infusion ends, and the adaptive controller takes over drug delivery. We also highlight the trend seen in data by plotting a smoothed curve (blue lines) obtained by passing the raw data (black dots) through first a Hampel filter over 10 min interval and second moving average smoothing filter over a 5 min interval.

The adaptive controller can also be used to rapidly and accurately follow pre‐defined, time‐varying concentration profiles. To show this, we explored the ability of the algorithm to follow two, pre‐defined stair‐step profiles. In the first, we raised the concentration to 100μM (which required 31.6 min), held it at this level (RMSD 4.9 μM) for 31 min, and then reduced the set point to 50μM, holding this (RMSD 4.1 μM) for an additional 38 min (Figure 5c). In the second, we stepped the targeted concentration in the opposite direction (Figure 5d). That is, we first targeted a concentration of 50μM, which was achieved after 32.6 min and held (RMSD 6.0 μM) for 41.7 min. We then raised the set point concentration to 100μM, achieving this after an additional 16.2 min, and held it (RMSD 11.3 μM) for about 1 h before ending the experiment.

The above attributes notwithstanding, of course, as currently implemented adaptive control is not without limitations. For example, while an ARMA (2, 1) model is shown to be a reliable approximation of the intercranial pharmacokinetics of procaine, other drugs and/or tissues may require more advanced models. Given this, exploring the sensitivity of our approach to changing pharmacokinetic conditions remains a potentially fruitful future research direction.

## 3 CONCLUSIONS

The real‐time in vivo molecular concentration information provided by EAB sensors provides a unique ability to perform fully autonomous, high‐precision feedback‐controlled drug delivery. Previously, we have exploited this using simple PID control algorithms designed using population pharmacokinetic models.^10^, ^12^, ^13^ These, however, rely on conservative tuning to prevent dangerous overshooting of the set point. Here, in contrast, we have developed an adaptive feedback controller that circumvents this problem, thus more rapidly achieving and more precisely maintaining constant or pre‐defined, time‐varying in vivo drug concentrations. It does so by dynamically learning the pharmacokinetics of drug distribution in each individual, without relying on any prior individualized or population‐level pharmacokinetic knowledge. This ability to rapidly and accurately control drug concentrations even in the face of large inter‐ and even intra‐ (i.e., over time^28^) pharmacokinetic variability could significantly improve the safety and efficacy of drug delivery. Adaptive feedback control could also hold considerable potential in the drug development arena. The ability to accurately mimic human pharmacokinetic time courses in animal models,^12^ for example, could improve the value of these models in drug development, potentially reducing the time and cost associated with bringing new therapies to market.

## 4 MATERIALS AND METHODS

### 4.1 Chemical reagents and materials

All reagents were purchased from Sigma‐Aldrich (St. Louis, MO) and used as received. The synthesis of the HPLC‐purified, reporter‐and‐thiol‐modified, procaine‐binding aptamer was obtained from Biosearch Technologies (Novato, CA), dissolved in deionized water to 100 μM, aliquoted into separate tubes, and stored at −20°C until used.

In‐brain procaine measurements were performed as previously described.^10^ In brief, in‐brain “probes” consisting of the sensor itself, an indwelling cannula, and a flexible leash were custom constructed by Plastics One (Roanoke, VA). The indwelling cannula, which also served as the counter electrode and a pseudo‐reference, was constructed from an 18‐gage stainless steel cannula (2 mm length below pedestal) with a plastic screw‐top connector (total length 13 mm). The sensor was constructed from a 22‐gage threaded stainless steel cannula (15 mm length below pedestal) filled with Teflon‐coated gold wire (76 μm diameter, 3 mm exposed, active sensor length, 19 mm total length), and surrounded by a plastic screw‐top connector with two ports for electrode pins. The electrode leash (50 cm) was built to contain male and female ends for electrode pins on opposite sides and is protected with a stainless‐steel mesh exterior.

To fabricate sensors, we first cleaned the gold wires by electrochemically with the following protocol: (i) 900 cycles between −1 and −1.6 V in a solution of 0.5 M NaOH at 1 V/s to remove any residual thiol/organic contaminants on the electrode surface and (ii) pulsed between 0 and 2 V for at least 16,000 cycles with a pulse length of 20 ms in 0.5 M H_2_SO_4_ to increase the electrode roughness. To prepare the aptamer for attachment to the gold surface we first reduced 4 μL of a 100 μM solution of the appropriate thiol‐and‐methylene‐blue‐modified oligonucleotide (5′‐thiol‐C6–PO_4_–AGAC AAG GAA AAT CCT TCA ACG AAG TGG GTCG–PO_4_–methylene blue‐3′) using 4 μL of 10 mM of tris(2‐carboxyethyl)phosphine hydrochloride (TCEP) for 1 h at 25°C. We then diluted the solution to 500 nM with 1× PBS (Phosphate‐Buffered Saline). We then submerged freshly cleaned electrodes in this solution for 1 h before moving them to a 10 mM 6‐mercaptohexanol solution overnight to form a self‐assembled monolayer.

For sensor interrogation we employed “signal‐on” and “signal‐off” square wave frequencies of 150 and 30 Hz, respectively. Using serial voltametric measurements performed at these frequencies we corrected the baseline drift seen in vivo using “kinetic differential measurements” (KDM).^9^ To convert KDM signals into drug concentrations we fitted the in vitro titration data to the following Langmuir equation (Equation 2).

(2) $$y=B_{\max}\times \frac{\left[\text{target}\right]}{K_{D}+\left[\text{target}\right]},$$

where y is the KDM signal, $B_{\max}$ is the signal change seen at saturating target concentrations, $\left[\text{target}\right]$ is drug concentration, and $K_{D}$ is the dissociation constant of the aptamer.

### 4.2 Animal housing, care, and surgery

Adult male Sprague–Dawley rats (350–600 g) obtained with a jugular catheter already emplaced (Charles River, Hollister, CA) were pair‐housed in a standard light cycle room (08:00 a.m. on, 20:00 p.m. off) and allotted ad‐libitum access to food and water. The housing and care of all rats were conducted in accordance with the guidelines set forth by the “Guide for the Care and Use of Laboratory Rats, 8th edition.^31^” We induced anesthesia prior to surgery using 4% isoflurane gas (Medline, Northfield, IL) and maintained it using 2%–3% for the remainder of surgical procedures. After inducing anesthesia, we shaved the head of the rats and disinfected the surgical site with 10% betadine solution and 70% isopropanol before injecting the local anesthetic lidocaine (2 mg/kg subcutaneous) into each incision site. Briefly, we implanted a 19‐gage stainless‐steel guide cannula (Plastics One, Roanoke, VA) and 22‐gage stainless‐steel obturator into the lateral ventricle (Anteroposterior (AP) = −0.48, Mediolateral (ML) = +1.6, Dorsoventral (DV) = −1). Temperature, heart rate, SPO2 (specific peripheral oxygen concentration), and respiration rate were recorded and maintained within veterinary recommended levels throughout surgical procedures. After surgery, we allowed the rats to recover for at least 7 days before experimentation. Following the experiment, we removed the brains from the rats and performed histology to confirm sensor placement, as described previously.^10^

### 4.3 Feedback‐controlled drug delivery

Before commencing measurements, we weighed each rat and induced brief (<5 min) anesthesia using 3% isoflurane gas, and quickly inserted the sensor through the cannula and affixed it in place by the screw fittings on the surgically implanted 19‐gage guide cannula. We then allowed the animal to recover from anesthesia and commenced EAB measurements using square‐wave voltammetry on a CH1208C mini‐potentiostat, alternating between square‐wave frequencies of 30 Hz (swept at 0.02 V/s) and 150 Hz (swept at 0.15 V/s), both at an amplitude of 25 mV and over a voltage window of approximately 0 to −0.45 V (relative to the stainless‐steel pseudo‐reference). To allow rapid drug delivery (IV), we employed chronic, indwelling jugular catheters (surgeries performed by Charles River). This was connected to a KDS230 syringe pump (Kd Scientific, Holliston, MA), the output of which was controlled by the feedback control algorithm.

### 4.4 Feedback control algorithm

Our algorithm comprises of solving two distinct yet related problems: (i) model identification problem and (ii) the optimal control problem. First, the controller identifies the optimal ARMA (2, 1) model by solving the following model identification problem formulated as a nonlinear optimization problem with equality constraints:

(3a) $$\min_{\alpha_{1},\alpha_{2},\beta_{0},\beta_{1},x}\sum_{i=t-N}^{t}{\left(y\left(i\right)-x\left(i\right)\right)}^{2},$$

(3b) $$\text{subject to}\;x\left(i\right)=\alpha_{1}x\left(i-1\right)+\alpha_{2}x\left(i-2\right)+\beta_{0}u\left(i-1\right)+\beta_{1}u\left(i-2\right),$$

where $\alpha_{1},\alpha_{2}$ are autoregressive coefficients, $\beta_{0},\beta_{1}$ are the moving average coefficients, $x\left(t\right)$ is the estimated noise‐free, and the $y\left(t\right)$ is the measured, but noisy, concentration at time t. The advantage of including dynamics as equality constraints, as in Equation (3b), lies in computational efficiency when using second order methods to solve the optimization (Equation 3a). The equality constraints enable this efficiency by making the Hessian of the problem much sparser.^32^

Second, the “optimal control problem” can be formulated as the following optimization that seeks for a control profile $u\left(t\right)$ that minimizes the squared error between the predicted concentration $x\left(t\right)$ and the desired reference concentration $r\left(t\right)$ over a time “future” time horizon from time t+1 to time t+M:

(4a) $$u^{*}=\underset{u}{\text{argmin}}\sum_{i=t+1}^{t+M}{\left(r\left(i\right)-x\left(i\right)\right)}^{2},$$

(4b) $$\;\text{subject to}\;x\left(i\right)=\alpha_{1}x\left(i-1\right)+\alpha_{2}x\left(i-2\right)+\beta_{0}u\left(i-1\right)+\beta_{1}u\left(i-2\right),$$

(4c) $$0\leq u\leq u_{\max},$$

where the ARMA model parameters ($\alpha_{1},\alpha_{2},\beta_{0},\;\text{and}\;\beta_{1}$) and the initial noise‐free concentration ($x\left(t\right),x\left(t-1\right)$) are obtained by the first identification stage formulated by Equations (3a and 3b). Once this optimal control problem has been solved, the control algorithm computes the optimal control input $u^{*}$ for the next M time intervals. This means that $u^{*}$ is the best course of action to take for the identified model for the next M time intervals. Both optimizations in problems (Equations 3a, 3b, 4a, 4b, and 4c) were solved using IPOPT's (Interior Point OPTimizer) Interior Point Method^33^, ^34^ accessed through CasADi's MATLAB interface.^35^

## AUTHOR CONTRIBUTIONS

Conceptualization: **MKE**, **KWP**, **JPH**. Methodology: **MKE**, **JG**. Investigation: **MKE**, **JG**. Visualization: **MKE**. Supervision: **TEK**, **KWP**, **JPH**. Writing—original draft: **MKE**, **KWP**. Writing—review and editing: **MKE**, **JG**, **TEK**, **KWP**, **JPH**.

## FUNDING INFORMATION

National Institutes of Health grant AI145206 (Murat Kaan Erdal, Julian Gerson, Tod E. Kippin, Kevin W. Plaxco, and Joao P. Hespanha).

## CONFLICT OF INTEREST STATEMENT

Kevin W. Plaxco owns equity in and consults for a company that is commercializing in vivo electrochemical aptamer‐based (EAB) sensors. Following the completion of this work, Murat Kaan Erdal and Julian Gerson became employees at a company that is commercializing in vivo EAB sensors. All authors are also co‐inventors in a patent application regarding the technology described in the manuscript.

## Supporting information


**Data SI.** Supporting Information.

## Data Availability

The data that support the findings of this study are available from the corresponding author upon reasonable request.
