# Supplementary material for: Adaptive feedback control for the high‐precision management of in‐tissue drug concentrations
Source: Bioeng Transl Med. 2026 Jun 3;11(5):e70146. doi: 10.1002/btm2.70146 (PMC13636143; doi:10.1002/btm2.70146)
Supplement: Supplementary file 1 [file BTM2-11-e70146-s001.docx]

Supplementary Information: Adaptive feedback control for the high-precision management of in-tissue drug concentrations

Murat Kaan Erdal Julian Gerson Tod E. Kippin, Kevin W. Plaxco, and Joao P. Hespanha

**Figure SI-1**. Shown are the data sets that form the population pharmacokinetics we use to tune our prior PID controller. The fitted lines are matched with the simulated responses in **Figure 2A, Figure 2C** and **Figure 2D**.

**Algorithm SI-1.** Adaptive Model Predictive Algorithm

Initialization Step: start with $r=50$,

While concentration is less than $C_{1}$,

Inject at maximum infusion rate

While concentration is less than $C_{2}$,

Inject at $r$ percent of the maximum infusion rate,

If we have been injecting at the same rate for $T_{update}$ min,

$r=\max\left( 1.5\times r,100 \right)$

Adaptive Control Steps:

While target concentration is nonzero,

Estimate the approximate ARMA (2, 1) with $\alpha^{*},\beta^{*}$ by solving (Eq 3)

Using $\alpha^{*},\beta^{*}$, find the optimal control $u^{*}$ by solving (Eq 4)

Apply the first value of $u^{*}$

Update time $t = t+1$
